# Stable isotope analysis of atmospheric CO_2_ using a Gasbench II‐Cold Trap‐IRMS setting

**DOI:** 10.1002/rcm.9647

**Published:** 2023-10-24

**Authors:** Simon Leitner, Kathiravan Meeran, Andrea Watzinger

**Affiliations:** ^1^ Institute of Soil Research University of Natural Resources and Life Sciences, Vienna Tulln Austria

## Abstract

**Rationale:**

The measurement of the stable carbon and oxygen isotope ratio of (atmospheric) carbon dioxide (CO_2_) is a useful technique for the investigation and identification of the sources and sinks of the most abundant greenhouse gases by far. For this reason, we are presenting a measuring system here that enables a wide range of users to carry out stable isotope analysis of atmospheric CO_2_ using off‐the‐bench hardware and software.

**Methods:**

The fully automated system uses cryogenic and gas chromatographic separation to analyse CO_2_ from 12‐mL whole air samples and consists of an autosampler, a Gasbench II (GB), a downstream cryo trap and a continuous flow gas interface feeding into a sector field mass spectrometer (GC Pal/GB/Cold Trap/ConFlo IV/DeltaV Plus). The evaluation of the system performance was based on the analysis of samples prepared from eight CO_2_ sources (four CO_2_ reference gases and four artificial air tanks).

**Results:**

The overall measurement uncertainty (averaged single standard deviation (1σ) of measurement replicates from each CO_2_ source) in the determination of the carbon and oxygen isotope ratio was 0.04‰ and 0.09‰ (*n* = 24). Furthermore, we were able to show that the measurement data also allowed for the quantification of the CO_2_ mole fraction, with a precision of 1.2 μmol mol^−1^ in the analysis range of 400–500 μmol mol^−1^.

**Conclusions:**

Our protocol provides a detailed description of the measurement set‐up and the analysis procedure, how raw data should be evaluated and gives recommendations for sample preparation and sampling to enable a fully automated whole air sample analysis. The quantification limit of CO_2_ mole fractions and measurement precision for carbon and oxygen isotope ratios of CO_2_ should meet the requirements of a wide range of users.

## 1 INTRODUCTION

The industrial era led to a change in the proportion of greenhouse gases in the atmosphere, with carbon dioxide (CO_2_) currently accounting for about 66% of the global warming potential (2021 global mean abundance of CO_2_: 415.7 ± 0.2 μmol mol^−1^).^1^ To take mitigation measures, it is necessary to know not only the emission levels but also their sources and sinks.^2^ The stable isotope ratios of carbon and oxygen in CO_2_ can be linked to the processes of its sources and sinks (e.g., burning of fossil fuels, photosynthesis, respiration, exchange reactions). The latter, (^13^C/^12^C, ^18^O/^16^O), can be determined using stable isotope ratio mass spectrometry (IRMS) or laser‐absorption spectroscopy (LAS).^3^ As far as IRMS techniques are concerned, most published systems rely on hand‐made peripherals, high instrumental know‐how or do not allow fully automated sample analysis. See the current review of Manaj and Kim (2020).^4^ Although these systems achieve high measurement accuracy and a high sample throughput, they have been individually adapted or optimised, which can be implemented only by experienced technical personnel and using specific components.

Here, we present a protocol on how to set up and operate an off‐the‐shelf continuous flow (CF)‐IRMS measurement system to analyse the carbon and oxygen stable isotope ratio of CO_2_ from whole air samples at ambient atmospheric mixing ratios. The aim of the presented measurement set‐up was to provide a fully automated system, including sample vessel preparation, sampling and sample analysis, which provides reasonable measurement performance and is available to a broad user community. The targeted measurement precision was <0.1‰ and <0.2‰ for the carbon and oxygen isotope ratio of CO_2_, respectively. Furthermore, it was tested whether the measurement set‐up would also allow for the estimation of the CO_2_ mole fraction in whole air samples.

The CF measurement set‐up presented here is based on the extraction of CO_2_ from 12 mL sample vessels using cryo‐focusing and gas chromatography. Therefore, a Gasbench II (GB; Thermo Fisher Scientific, Bremen, Germany) equipped with a 250‐μL sample loop and a subsequent Cold Trap was connected to a CF gas distribution system (ConFlo IV, Thermo Fisher Scientific, Bremen, Germany) managing the gas inlet into a sector field isotope ratio mass spectrometer (Delta V Plus, Thermo Fisher Scientific, Bremen, Germany). The measurement uncertainty was evaluated by the analysis of four distinct CO_2_ reference gases with assigned true isotope ratios mixed with synthetic air for the analysis. The measurement set‐up was then further used to calibrate the isotope ratios of CO_2_ in four artificial atmospheric air tanks to be used as working gases in atmospheric air monitoring campaigns. Furthermore, the impact of cryo‐focusing, chromatographic separation, water background levels and the open‐split sample dilution setting (‘blanking’) were evaluated and are discussed below.

## 2 MATERIALS AND METHODS

### 2.1 CF‐IRMS set‐up

**FIGURE 1:**
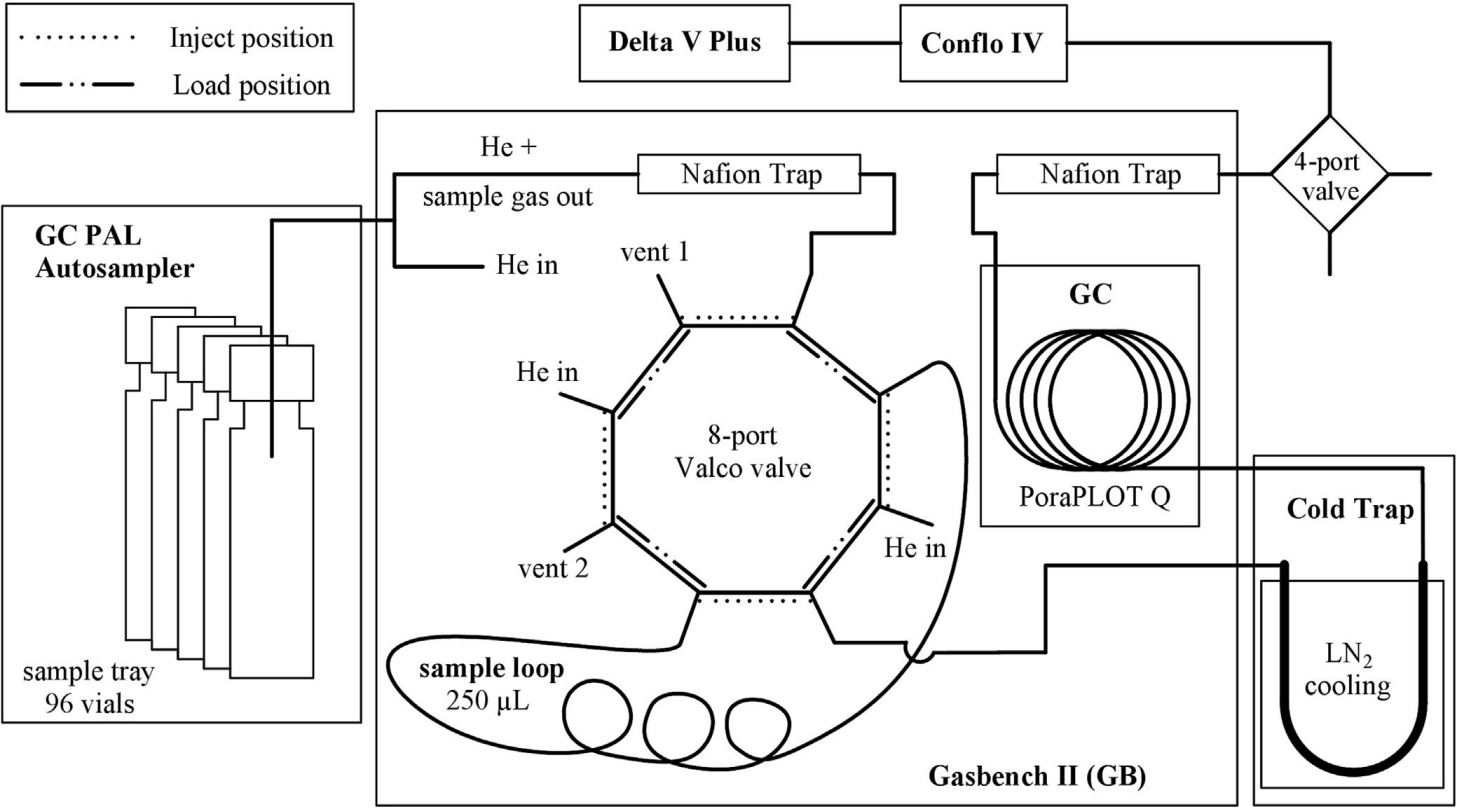
Instrumental set‐up for the analysis of 12‐mL whole air samples using a GC PAL autosampler and a Gasbench II (GB) connected to a Cold Trap, a ConFlo IV and a Delta V Plus.

**FIGURE 2:**
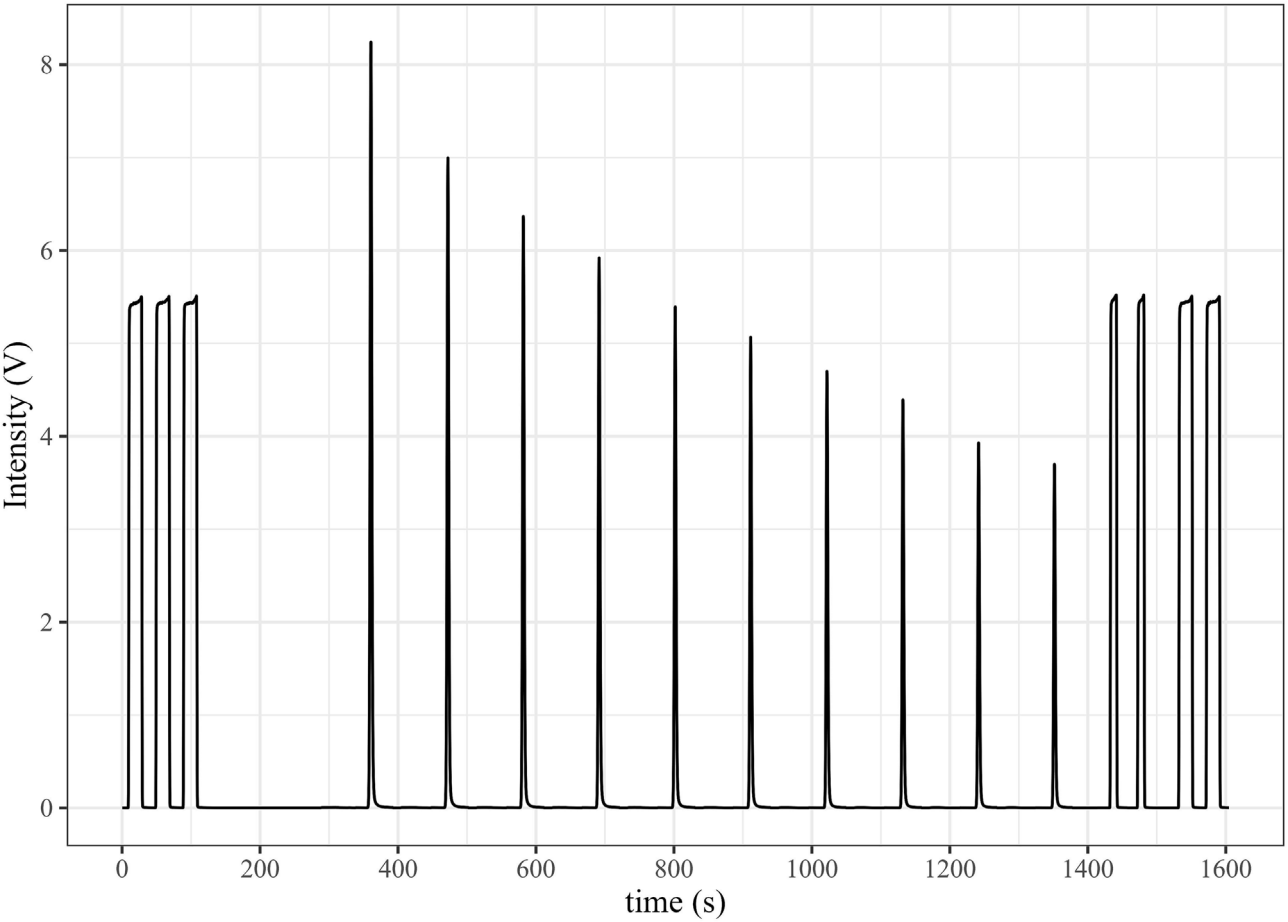
Chromatogram for the analysis of CO_2_ from a 12‐mL whole air sample (B1 air tank; CO_2_: 404 μmol mol^−1^) and the recording of the mass‐44 signal intensities. Rectangular peaks represent CO_2_ working gas peaks, and Gaussian‐shaped peaks represent CO_2_ sample gas obtained from 250‐μL sample gas aliquots.

The measurement set‐up consisted of a GC PAL Autosampler (CTC Analytics AG, Zwingen, Switzerland), a GB, a Dual Cold Trap (Thermo Fisher Scientific, Bremen, Germany), using the fused silica capillary trap only, a ConFlo IV and a Delta V Plus. The ensemble is shown in Figure 1. The autosampler was equipped with a heating unit (25°C) and a sample tray for 96 sample vials containing 12 mL Exetainer glass vials (Labco Limited, Lampeter, UK) and was only used to manoeuvre the double needle, which directs the sample gas from the sample vial to the GB at ~0.8 mL min^−1^. Alternatively, the set‐up could also be used with 20 mL headspace vials with crimped polytetrafluoroethylene (PTFE)‐coated butyl rubber septa, as presented in Leitner et al (2020).^5^, ^6^ At the GB (helium [He] inlet pressure of 80 kPa), the sample gas was first dried by passing through a Nafion™ membrane (purged continuously with a counter current stream of He), was then sent through a 250 μL sample loop and eventually vented from the system. At an initial transfer time of 160 s, the sample loop was further flushed for 60 s before switching the eight‐port valve from the ‘Load’ position to the ‘Inject’ position to transfer the sample loop volume to the Cold Trap, which had been immersed in a 3‐L liquid nitrogen (LN_2_) dewar already 10 s ahead. The sample loop was flushed for 50 s to ensure complete transfer of sample gas to the Cold Trap before switching the eight‐port valve back to the ‘Load’ position. The Cold Trap was released 10 s thereafter to transfer the cryo‐focused gases (CO_2_, N_2_O, water vapour) and residual air to a 25 m × 0.32 mm PoraPLOT Q GC‐column (Agilent Technologies GmbH, Vienna, Austria) to separate CO_2_ from the other air constituents. The gas chromatography (GC) outlet was linked to a second Nafion™ trap before entering the sample open split via the low‐flow capillary inside the gas distribution system (ConFlo IV), which then transferred the sample gas to the IRMS (Delta V Plus), as shown in Figure 1. To further dilute residual N_2_ and O_2_, the mass spectrometer (MS) capillary inside the ConFlo IV sample open split was connected only shortly before and during the retention times of CO_2_ from the 250‐μL gas samples. This procedure of loop filling, trapping and CO_2_ peak detection was repeated 10 times during the total analysis time of 30.2 min per sample vial. Detailed event timings are provided in Table S1 (supporting information). The monitoring of the CO_2_ sample gas peaks was performed together with CO_2_ working gas peaks (rectangular peaks, fixed mass 44 intensity of 5 V) added before and after the set of sample gas peaks (Figure 2). As the IRMS is also used to analyse samples by GC, a manual four‐port valve was placed in front of the low‐flow inlet of the ConFlo IV to switch between GB and GC.

The impact of temperature and pressure effects on the IRMS magnet performance, as pointed out by Ferretti et al (2000),^7^ was taken care of by the use of an air conditioning system inside the lab maintaining the lab air temperature at 22°C.

### 2.2 Sample vial and air tank preparation

Gas was sampled in 12‐mL Exetainer glass vials (Labco Limited, Lampeter, UK) sealed with grey chlorobutyl septa and screw caps. Vials were prepared by flushing with synthetic air (SA; synthetic air 5.0 HC‐free, Messer Austria GmbH, Gumpoldskirchen, Austria), which refers to zero‐air, for 60 s at a flow of 250 mL min^−1^. Different settings of the inlet pressure, flush time and flush gas flow were tested, and we recommend exchanging the sample vial volume for at least 20 times to obtain CO_2_‐free vials. Flushing was performed using two G26 side‐bore needles piercing the septum of the Exetainer vial with the cap closed. For the preparation of isotope reference gas standards, 5 μL of four pure CO_2_ reference gases (R1, R2, R3, R4; ISO‐TOP, Messer Austria GmbH) and the CO_2_ working gas were added on top of the SA‐flushed vial using a 10‐μL gas‐tight syringe equipped with a G26 side‐bore needle (Hamilton Bonaduz AG, Bonaduz, Switzerland).

Whole air samples were mimed by using four 50‐L air tanks (7–10 MPa, Messer Austria GmbH), labelled B1, B2, B3 and B4, which had been prepared by adding pure CO_2_ to 50‐L SA tanks. The SA used to flush the Exetainer vials and that used to manufacture the air tanks was of the same quality and composition and consisted of a 79.5:20.5 mixture of N_2_ and O_2_ and the minor components CO_2_ (≤0.5 μmol mol^−1^), NOx (≤0.1 μmol mol^−1^) and H_2_O (≤5 μmol mol^−1^). The final isotopic composition of B1, B2, B3 and B4 was varied by the admixed amount of CO_2_ added from another two pure CO_2_ cylinders containing ^13^C‐depleted or ^13^C‐enriched CO_2_. The four air tanks obtained four distinct isotopic compositions and mole fractions of CO_2_, similar to the levels expected for atmospheric air (410–520 μmol mol^−1^, δ^13^C: −8 to −18‰).^8^, ^9^, ^10^, ^11^, ^12^, ^13^, ^14^, ^15^, ^16^ The mole fraction of CO_2_ was 404, 404, 513 and 406 μmol mol^−1^ ± 2% absolute deviation, according to the Messer company, for B1, B2, B3 and B4, respectively. Whole air samples were prepared by flushing the Exetainer vials with tank air similar to the setting as that for SA‐flushing, but after the vials had been flushed with SA initially. For whole air sampling of atmospheric air, we refer to the sampling procedure presented in the recent publication of Leitner et al (2020 and 2023).^5^, ^6^

### 2.3 Referencing of carbon and oxygen isotope ratios of CO_2_

The stable carbon and oxygen isotope ratio of CO_2_ is reported in the δ‐notation (‰) and was referenced to the Vienna Peedee Belemnite (VPDB) scale for δ^13^C and δ^18^O values, anchored by isotope reference materials and internally cross‐checked (data not shown) by the analysis of CO_2_ liberated by H_3_PO_4_ acid digestion of NBS18 (δ^13^C: −5.014‰ ± 0.035 vs VPDB, δ^18^O: −23.2‰ ± 0.1 vs VPDB)^17^ to be sure about the reference scale given for the isotope reference gases described below. In Brand et al,^18^ there is a recommendation that carbonate reference materials, specifically NBS19, should not or not exclusively be used as δ^18^O reference anchors in the analysis of CO_2_ gases if these gases were obtained without acid digestion (e.g., CO_2_ in air). δ‐values were calculated as

$$\delta^{13}C=\frac{R_{P}}{R_{\mathrm{Std}}}-1\;\delta^{18}O\;=\frac{R_{P}}{R_{\mathrm{Std}}}-1\;,$$

where *R* is the ratio of the abundance of ^13^C to ^12^C and ^18^O to ^16^O of a sample (P) and a measurement standard (Std).^19^

Four certified CO_2_ reference gases (R1, R2, R3, R4; ISO‐TOP, Messer Austria GmbH) with an assigned true δ^13^C value versus VPDB of −6.65 ± 0.13‰, −6.73 ± 0.13‰, −25.71 ± 0.20‰ and −39.00 ± 0.21‰ and assigned true δ^18^O value versus VPDB of −17.82 ± 0.20‰, −17.97 ± 0.20‰, −28.72 ± 0.15‰ and −30.34 ± 0.20‰ were used to determine the isotopic composition of the CO_2_ in four 50‐L air tanks, labelled B1, B2, B3, B4. The four 50‐L air tanks were prepared by admixing CO_2_ from two pure CO_2_ cylinders filled with a ^13^C‐depleted and a ^13^C‐enriched CO_2_ gas, not related to the four certified CO_2_ reference gases. The CO_2_ working gas (δ^13^C: −4.29‰ ± 0.13‰; δ^18^O: −12.27‰ ± 0.17‰; *n* = 7) of the IRMS was also referenced with the use of the certified CO_2_ reference gases, but via direct injection to a GC/IRMS set‐up as presented at Leitner et al.^6^ Monitored working gas peaks, added before and after the sample gas peaks, indicated a single standard deviation (1σ) of δ^13^C‐ and δ^18^O‐values over the course of the presented measurements (*n* = 255) of 0.08‰ and 0.06‰, respectively.

### 2.4 Evaluation of CO_2_ measurement data

The measurement data presented include the analysis of three sample vials per air tank and per reference gas, the latter being added to vials filled with synthetic air. The sample vials were analysed in a single sequence run. Analysis of each sample vial comprised 10 CO_2_ gas peaks obtained by continuous filling and subsequent transfer of a 250‐μL sample loop volume. As a result of the dilution of the sample gas with the carrier gas (He), there was a decrease in the mass intensities (~60% between the first and tenth CO_2_ peaks). Therefore, raw data were first corrected for the δ ^13^C and δ^18^O non‐linearity effect because of the dependence of mass 44, 45 and 46 signal intensities. Non‐linearity corrected peaks of each sample vial were checked for outliers using a Grubbs Test^20^ to then decide on the most optimal number of peaks to be used to calculate a sample’s mean value of δ ^13^C and δ^18^O. Then, δ‐values were normalised with the use of those samples, which had been prepared with the four reference gases, using linear regression and according to Paul et al.^21^ Analysed samples comprised three sample replicates of each CO_2_ source (R1, R2, R3, R4, B1, B2, B3 and B4), analysed in a single sequence run. Normalised data were grouped by CO_2_ source to obtain a final calibrated mean value ± 1σ (measurement uncertainty) and combined uncertainty (u) for the air tanks (B1, B2, B3, B4). The initial raw data were obtained by the software Isodat (version 3.0) from Thermo Fisher Scientific, which is also used to operate the measurement system presented. For information on peak detection parameters and δ‐value calculation, see the Supporting Information.

## 3 RESULTS AND DISCUSSION

### 3.1 Chromatographic resolution

An exemplary chromatogram for the analysis of CO_2_ in 12‐mL whole air samples using the presented method is shown in Figure 2. It shows the intensity of mass 44 while analysing a sample vial containing 404 μmol mol^−1^ CO_2_ and rectangular working gas peaks before and after the 10 CO_2_ sample gas peaks. Sample gas peaks continuously decrease in time due to the mixing with the He carrier gas inside the sample vial. Each sample peak represents the CO_2_ mole fraction in 250‐μL sample gas and, at a CO_2_ mole fraction of 400 μmol mol^−1^, was initially at ~8.0 V and decreased to ~3.6 V within 10 sample gas peaks.

**FIGURE 3:**
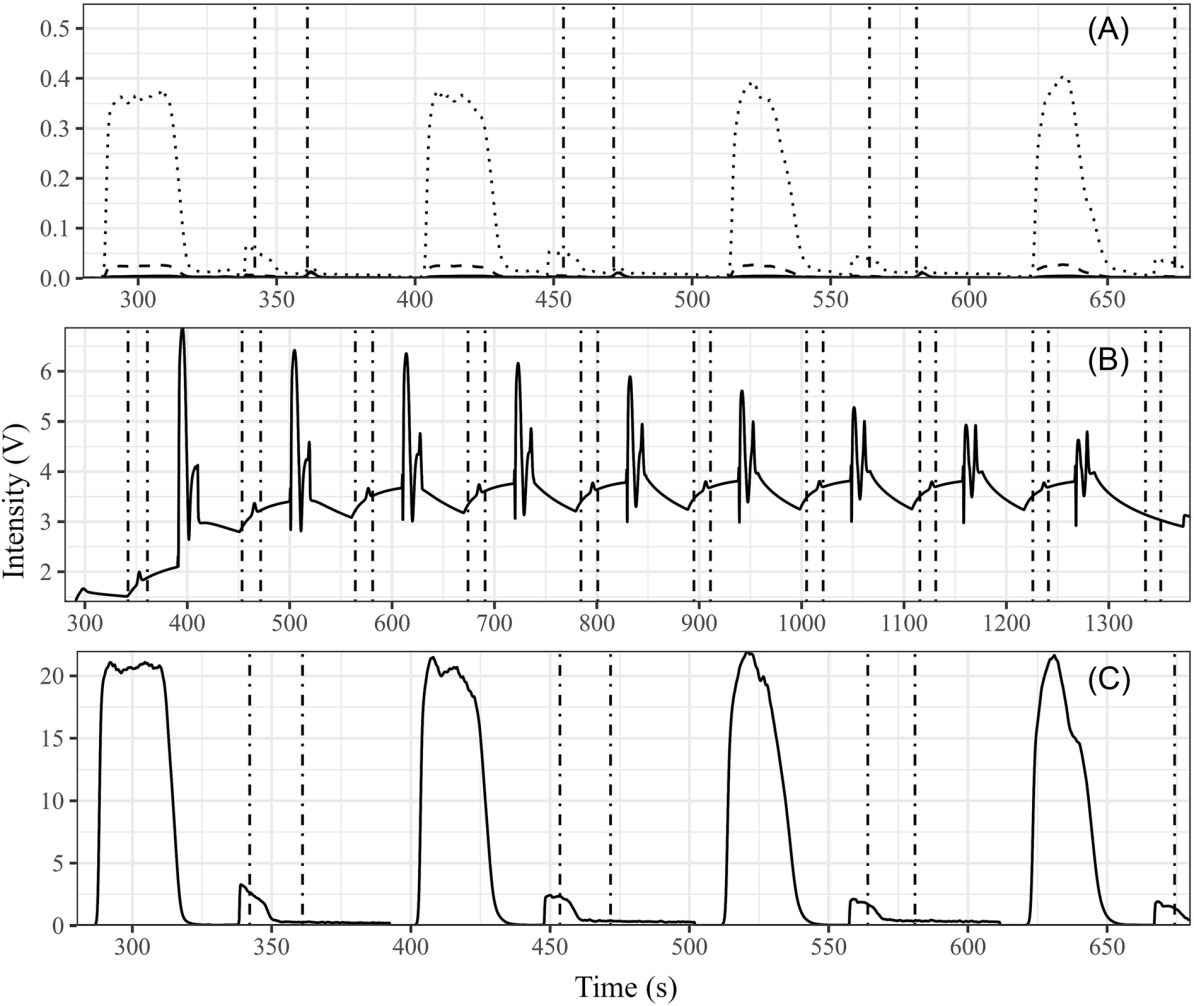
Results from the measurement of a blank vial (filled with synthetic air [SA] only) shown in (A) and (C) and of a vial filled with lab air in (B). Vertical dot‐dashed lines indicate the position of CO_2_ sample gas peaks (retention time ± ½ the width). Mass scans comprised mass 44, 45 and 46 indicated by solid, dashed and dotted lines (A), mass 18 (B) and mass 28 (C).

Mass intensities obtained from a blank‐sample measurement, that is, a sample vial purged with SA without the addition of pure CO_2_, are shown in Figure 3. The intensity of mass 44 ranged from 30 to 15 mV from the first to the tenth sample gas peaks and was considered negligible for evaluation (shown for peak 1:4 in plot A of Figure 3). Isobaric interference from residual N_2_ and O_2_ in the ion source cavity could not be identified from mass‐46 and mass‐28 scans, respectively, as shown in plot A (dotted line) and plot C (solid line) of Figure 3. The water background, which affects the mass 45 and hence the carbon isotope ratio, is shown in plot B of Figure 3 and was tested by analysing laboratory room air‐filled measurement vials. Despite the initially increasing mass‐18 intensity (1.8 to 3.5 V for the first to third peaks (scanned on Faraday collector cup 3 [3 10^10^ Ohm]) and subsequent constant intensity, the CO_2_ retention times were always found at constant mass 18 background intensities. Therefore, they were not affected by the evaporation of the water previously frozen out in LN_2_, as this only occurred ~20 s after each CO_2_ peak.

### 3.2 Raw data processing

#### 3.2.1 Non‐linearity correction and identification of outliers

Each sample analysis provided 10 carbon and oxygen isotope ratio values that should be corrected for the non‐linearity effect of the IRMS detector due to the decrease in mass intensities. The manufacturer recommends correcting the δ‐values using linear regression (first‐order polynomial function) of δ‐value and mass‐44 intensity, which must be generated from separate IRMS linearity measurements and should be performed before each measurement sequence. For δ^13^C, the slope from the linear regression was found to be 0.06‰ mV^−1^ (0.02‰ nA^−1^) with a coefficient of determination (*R* ^*2*^) of 0.91. It was found in our laboratory that regression using a second‐order polynomial function better described (*R* ^*2*^: >0.99) the relation between δ^13^C and mass‐44 intensity (Table S2 and Figure S1 [supporting information]). Similarly, the non‐linearity effect in the oxygen isotope ratio did not indicate a linear dependency on the mass intensity and showed a fluctuation in δ^18^O‐values of ±0.02‰ at most and was therefore not taken into account (Figure S1). The circumstance of the observed non‐linearity effects could have been due to the condition of the ion source components or the low residual water content in the He gas and its influence on the ionisation and should therefore be treated individually. As a point of reference, it should be noted that the intensity of mass 18, scanned at the Faraday cup 3, was often less than 500 mV before the start of a measurement sequence, and this represents very ‘dry’ He.

A comparison of the δ^13^C single standard deviation per sample after non‐linearity correction, using first‐ and second‐order polynomial functions, as well as in comparison to raw δ^13^C‐values is shown in Figure S2 (supporting information) and illustrates that the use of a second‐order polynomial function was most effective to reduce the standard deviation of δ^13^C sample means.

After non‐linearity correction of the sample peaks, an outlier test of the δ^13^C and δ^18^O peak values of each sample was performed. Two outliers were found for carbon and three for oxygen within the data set of 240 values each (24 samples, 10 peaks each). These were distributed among individual samples and were considered random and non‐systematic outliers and therefore removed from the dataset.

#### 3.2.2 Number of CO_2_ peaks retained from single sample measurements

In the next step, the optimal number of sample peaks (Figure 2) used in the data processing was determined based on the variation of the δ^13^C and δ^18^O single standard deviation (1σ) of the sample means (top plot) and means per CO_2_ source (bottom plot) shown in Figures S3 and S4 (supporting information). The analysis of individual samples revealed no significant change in the median 1σ values with expanding the number of included CO_2_ sample gas peaks (*n*) from *n* = 3 to *n* = 10 (Figure S3 [supporting information]). However, there was a reduction in the interquartile range of the 1σ values with an increasing number of consecutive CO_2_ peaks used. The variation in 1σ values showed a similar picture for data grouped by CO_2_ source as for individual sample vials. In principle, we recommend including all 10 sample gas peaks in the evaluation, as the lowest 1σ for gas sample replicates can be achieved in this way (Figure S3, S4 [supporting information]). If the measurement accuracy of replicate samples is not a priority, or if only single samples are analysed, one could reduce the number of sample gas aliquots analysed to five to increase sample throughput and one could expect a probably slightly larger 1σ of the single sample mean value. Although the analysis of the oxygen data showed significantly higher standard deviations (Figure S4 [supporting information]), the picture compared to the carbon data was the same. We therefore recommend the same procedure for evaluating the oxygen data as for the carbon data. The 1σ value of the means using 10 CO_2_ sample gas peaks was better than ±0.14‰ and ±0.07‰ for δ^13^C single sample vials and for CO_2_ source means and better than ±0.60‰ and ±0.20‰ for those of δ^18^O.

#### 3.2.3 Normalisation and system performance

The δ‐values of 10 CO_2_ peaks per sample vial were averaged and denoted as mean values per sample vial. The mean values per sample vial of the CO_2_ reference gases (R1, R2, R3, R4) were used to normalise the δ‐values of the tank samples of B1, B2, B3, B4 by linear regression of the assigned true versus gathered δ‐values and according to Paul et al.^21^ The linear regression parameters showed a slope of 1.008 and 1.000 and an *R* ^*2*^ better than 0.999 for δ‐values of carbon and oxygen, respectively. The mean values of the CO_2_ reference gas sample vial were checked by normalisation with the assigned true δ‐values of the remaining three reference gases. The respective regression parameters always had a slope of 1.008 for carbon and between 0.99 and 1.01 for oxygen with an *R* ^*2*^ better than 0.999.

Normalised sample vial means were grouped by CO_2_ source (B1, B2, B3, B4, R1, R2, R3, R4) to obtain a calibrated mean δ‐value and single standard deviation (1σ) for each CO_2_ source (*n* = 3). Results are presented in Table 1 and indicate a measurement uncertainty for the analysis of CO_2_‐source sample replicates (single standard deviation [1σ]) of less than 0.07‰ and 0.20‰ for carbon and oxygen isotope ratios, with an overall mean precision of 0.04 and 0.09‰, respectively. The four air tanks serve as calibration gases in the monitoring of urban CO_2_ inventories using LAS.^22^ The specified 1σ represents the measurement uncertainty and uncertainty in the production of sample replicates. To include the uncertainty associated with the uncertainty of the δ‐values of the reference gases used for normalisation, the model of Meija and Chartrand^23^ was applied to calculate a combined uncertainty (u) for the δ‐values of each air tank, which is also presented in Table 1.

**TABLE 1:** Overview of the results obtained from the analysis of reference gases (R1, R2, R3, R4) admixed to synthetic air (SA) and samples from four air tanks (B1, B2, B3, B4).

CO_2_ source	Name	δ^13^C					δ^18^O					CO_2_ mole fraction			
		Assigned true	σ	Calibrated mean	1σ	u	Assigned true	σ	Calibrated mean	1σ	u	Calibrated	σ	Recovered mean	1σ
Reference gas	R1	−6.65	0.13	−6.62	0.04		−17.82	0.20	−17.59	0.05					
Reference gas	R2	−6.73	0.13	−6.76	0.06		−17.97	0.20	−18.17	0.14					
Reference gas	R3	−25.71	0.20	−25.71	0.02		−28.72	0.15	−28.89	0.13					
Reference gas	R4	−39.00	0.21	−39.00	0.07		−30.34	0.20	−30.14	0.06					
Air tank	B1			−14.65	0.03	0.13			−18.57	0.05	0.2	404	8.1	404.6	1.1
Air tank	B2			−10.08	0.06	0.10			−16.17	0.20	0.4	404	8.1	403.3	0.4
Air tank	B3			−17.56	0.03	0.13			−20.14	0.03	0.2	513	10.3	513.0	1.2
Air tank	B4			−7.60	0.02	0.12			−14.91	0.07	0.3	406	8.1	405.9	0.9
					0.04					0.09					

*Note*: Calibrated mean values (*n* = 3) represent normalised carbon and oxygen δ‐values (vs Vienna Peedee Belemnite [VPDB] using the assigned true values ±σ of R1, R2, R3, R4) ± the single standard deviation (1σ) representing the measurement uncertainty and the combined uncertainty (u) for the calibrated δ‐value means of B1, B2, B3 and B4. The calibrated mole fraction (± σ) of CO_2_ in B1, B2, B3 and B4 is presented versus the recovered mean mole fraction ±1σ.

In general, it is recommended to always follow the identical‐treatment‐principle^24^ of samples and reference materials when analysing CO_2_ from gas samples to avoid ambiguities about the calibrated isotope ratios obtained. For reference materials, we refer to Brewer et al.^25^ for a comprehensive review of appropriate reference materials to use when analysing gas samples (whole air samples), and to Ghosh et al.^26^ on scale anchoring. Identical treatment also accounts for matrix effects, as discussed in Levitt.^27^ With the method presented by us, the sample vials are flushed with synthetic air before either CO_2_ reference gas is admixed manually or tank air is flushed through. However, the remaining matrix (SA) in the vials was the same, as the SA used for flushing and making the air tanks was from the same manufacturer (Messer) and of the same quality. Furthermore, due to the slopes of the linear regression lines for the δ‐value normalisation, no matrix effect could be identified. In addition, the calibrated δ‐values of the reference gases did not indicate a larger offset for ^13^C‐depleted CO_2_, which Tu et al^28^ had been reported.

Apart from maintaining the identical treatment, the evaluation of the measurement set‐up has tried to also tackle other analytical problems arising from the analysis of gas (air) samples. Due to an N_2_/O_2_ sample gas matrix, problems caused by ‘blanking’ (relative position of the MS and the sample capillary of the open split of the low‐flow sample) as discussed in Elsig and Leuenverger^29^ must be prevented. Resulting isobaric interferences could not be identified according to the results presented in the section on chromatographic resolution. The problem of isotopic exchange with accumulated water carried over from the sample vials was investigated as follows. First, at evaluating the number of CO_2_ peaks from single sample measurements (Section 3.2.2), there was no indication of progressive change in the δ‐values of successive CO_2_ peaks. Second, with the determination of the maximum sample vial throughput before the LN_2_ trap has to be refilled. Therefore, a measurement sequence of sample vials filled with laboratory air was run. The automatic operation time limit was 23.7 h, and the drift of the δ‐values was 7.6 10^−6^‰ h^−1^ and 4.0 10^−5^‰ h^−1^ for δ^13^C‐ and δ^18^O, which also pointed towards minor water issues. Another factor, sample vial contamination (CO_2_ peaks obtained by analysis of sample vials flushed with SA only), also proved to be negligible (Figure 3). Finally, as the 1σ values did not depend on the magnitude of the calibrated δ‐values, memory effects (i.e., reminiscences of the previous sample that changed the isotopic composition of the measured sample) were not observed.

#### 3.2.4 Calculation of the CO_2_ mole fraction

The air tank sample measurement data (B1, B2, B3, B4) was also used to back‐calculate the CO_2_ mole fraction, based on the correlation of the sum of the CO_2_ peak area (Vs), including peaks 1 to 10, and the calibrated CO_2_ mole fraction according to Messer AG. The results of the linear regression showed that the data points were correlated with an *R* ^*2*^ of 1.00 and gave a *y*‐intercept of −17 μmol mol^−1^ when extrapolated through the origin. The calculated recovered CO_2_ mole fractions were found to have a maximum deviation from the calibrated values of 0.7 μmol mol^−1^ and gave a single standard deviation of CO_2_ source sample replicates of better than 1.2 μmol mol^−1^ (Table 1). Defining the limit of quantification as 20 times the mass 44 background intensity (30–15 mV at peaks 1 to 10), which also ensures a signal intensity within IRMS detector non‐linearity, would yield a minimum CO_2_ mole fraction of ~57 μmol mol^−1^.

## 4 CONCLUSIONS

The presented protocol describes the design and operation of a measuring system for the determination of the stable carbon and oxygen isotope ratio and mole fraction of CO_2_ in air. Compared to already‐existing measuring systems, it is characterised by the sole use of readily available system components. The system improved the standard installation of the measuring system GB/ConFlo IV/Delta V Plus from Thermo Fisher Scientific by using a 250‐μL sample loop in combination with a downstream Cold Trap. As a result, when measuring 400 μmol mol^−1^ CO_2_ in the air, the system showed CO_2_ peak amplitudes of 8 V and excellent peak separation from other air constituents (N_2_, O_2_, water). There was no indication of memory or matrix effects, isobaric interference, ‘blanking’ issues, δ‐value drift or instrumental and sample vial contaminations with lab air.

Although the described system could not reach the measuring precision of a dual inlet system (δ^13^C: 0.01‰, δ^18^O: 0.02), it allows a reliable, fully automated sample analysis (~30 min each), using small sample volume flasks (12 mL). The system was found to be capable of providing accurate δ^13^C‐ and δ^18^O‐values and mole fractions of CO_2_ in the air with an averaged measurement precision (1σ) of 0.04‰, 0.09‰ and 1.2 μmol mol^−1^, respectively. The measurement accuracy achieved should therefore allow the isotope ratios obtained to be used in Keeling plot applications,^30^, ^31^ to study ecosystem respiration, to monitor urban CO_2_ emissions and to determine CO_2_ sources and sinks. From a more general point of view, it can be used to estimate exchange reactions between the ecosystem and the atmosphere. Finally, we also see the potential for use in the referencing/normalisation of secondary air reference tanks for field applications.

## AUTHOR CONTRIBUTIONS

**Simon Leitner:** Conceptualisation (equal); formal analysis (lead); investigation (equal); methodology (equal); validation (lead); visualisation (lead); writing—original draft preparation (lead). **Kathiravan Meeran:** Investigation (equal); writing—review and editing (equal). **Andrea Watzinger:** Conceptualisation (equal); methodology (equal); writing—review and editing (equal).

### PEER REVIEW

The peer review history for this article is available at https://www.webofscience.com/api/gateway/wos/peer-review/10.1002/rcm.9647.

## Supporting information


**Table S1.** Time events of the Isodat isotope ratio mass spectrometry (IRMS) method entered in the time event tab. The Isodat configurator, therefore, needs to have the gas bench node added to the LF (low flow capillary) of the ConFlo IV interface, which has to be the source of the respective mass spectrometer (MS). The autosampler is added as AS2000 at the input of the gas bench node. The time event columns MS Capillary—ON and SamplDil 1—3 have to be added at the ConFlow IV interface events tab.
**Table S2.** Regression parameters for the δ‐value versus mass 44 amplitude isotope ratio mass spectrometry (IRMS) detector non‐linearity.
**Figure S1.** Plots of the isotope ratio mass spectrometry (IRMS)‐detector non‐linearity of the δ‐value and mass 44 amplitudes for carbon (top) and oxygen (bottom).
**Figure S2.** Comparison of the single standard deviation (1σ) of the δ^13^C values obtained from 10 carbon dioxide (CO_2_) peaks per sample dependent on the type of linearity correction applied to raw δ^13^C values. Points shaped as triangles show the difference between linearity corrected values using quadratic (*x*‐axis) and linear regression (*y*‐axis). Points shaped as rectangles show the comparison of 1σ calculated from raw δ^13^C values (*y*‐axis) versus quadratic regression corrected δ^13^C values (*x*‐axis).
**Figure S3.** Single standard deviation (1σ) of individual sample vials (top) and samples grouped by carbon dioxide (CO_2_)‐source (bottom) dependent on the number of CO_2_ measurement peaks used in the calculation of 1σ of carbon isotope ratios (δ^13^C) of CO_2_ in air.
**Figure S4.** Single standard deviation (1σ) of individual sample vials (top) and samples grouped by carbon dioxide (CO_2_)‐source (bottom) dependent on the number of CO_2_ measurement peaks used in the calculation of 1σ of the mean oxygen isotope ratio (δ^18^O) of CO_2_ in air.

## Data Availability

The data that support the findings of this study are available from the corresponding author upon reasonable request.
