# Supplementary material for: Stable isotope analysis of atmospheric CO2 using a Gasbench II‐Cold Trap‐IRMS setting
Source: Rapid Commun Mass Spectrom. 2023 Oct 24;37(24):e9647. doi: 10.1002/rcm.9647 (PMC10909479; doi:10.1002/rcm.9647)
Supplement: Supplementary file 1 — Table S1. Time events of the Isodat isotope ratio mass spectrometry (IRMS) method entered in the time event tab. The Isodat configurator, therefore, needs to have the gas bench node added to the LF (low flow capillary) of the ConFlo IV interface, which has to be the source of the respective mass spectrometer (MS). The autosampler is added as AS2000 at the input of the gas bench node. The time event columns MS Capillary—ON and SamplDil 1—3 have to be added at the ConFlow IV interface events tab. Table S2. Regression parameters for the δ‐value versus mass 44 amplitude isotope ratio mass spectrometry (IRMS) detector non‐linearity. Figure S1. Plots of the isotope ratio mass spectrometry (IRMS)‐detector non‐linearity of the δ‐value and mass 44 amplitudes for carbon (top) and oxygen (bottom). Figure S2. Comparison of the single standard deviation (1σ) of the δ13C values obtained from 10 carbon dioxide (CO2) peaks per sample dependent on the type of linearity correction applied to raw δ13C values. Points shaped as triangles show the difference between linearity corrected values using quadratic (x‐axis) and linear regression (y‐axis). Points shaped as rectangles show the comparison of 1σ calculated from raw δ13C values (y‐axis) versus quadratic regression corrected δ13C values (x‐axis). Figure S3. Single standard deviation (1σ) of individual sample vials (top) and samples grouped by carbon dioxide (CO2)‐source (bottom) dependent on the number of CO2 measurement peaks used in the calculation of 1σ of carbon isotope ratios (δ13C) of CO2 in air. Figure S4. Single standard deviation (1σ) of individual sample vials (top) and samples grouped by carbon dioxide (CO2)‐source (bottom) dependent on the number of CO2 measurement peaks used in the calculation of 1σ of the mean oxygen isotope ratio (δ18O) of CO2 in air. [file RCM-37-e9647-s001.docx]

Stable isotope analysis of atmospheric CO_2_ using a Gasbench II – Cold Trap – IRMS setting

Simon Leitner^1^, Kathiravan Meeran^1^, Andrea Watzinger^1^

^1^ University of Natural Resources and Life Sciences, Vienna, Institute of Soil Research, Konrad-Lorenz-Straße 24, 3430 Tulln, Austria

# Supporting Information

Table S1. Time Events of the Isodat IRMS method entered in the Time Event Tab. The Isodat Configurator therefore needs to have the Gas Bench Node added to the LF (low flow capillary) of the ConFlo IV Interface, which has to be the Source of the respective MS. The Autosampler is added as AS2000 at the Input of the Gas Bench Node. The Time Event columns MS Capillary – ON and SamplDil 1 – 3 have to be added at the ConFlow IV Interface Events tab.

| Time [s] | Reference Automatic | Valco Inject | Trap2 | MS Capillary - On | SampDil 1 - On | SampDil 2 - On | SampDil 3 - On |
| --- | --- | --- | --- | --- | --- | --- | --- |
| 0 | ON | OFF |  |  |  |  |  |
| 20 | OFF |  |  |  |  |  |  |
| 40 | ON |  |  |  |  |  |  |
| 50 |  |  | ON |  |  |  |  |
| 60 | OFF | ON |  |  |  |  |  |
| 80 | ON |  |  |  |  |  |  |
| 100 | OFF |  |  |  |  |  |  |
| 110 |  | OFF |  |  |  |  |  |
| 120 |  |  | OFF |  |  |  |  |
| 160 |  |  | ON |  |  |  |  |
| 170 |  | ON |  |  |  |  |  |
| 220 |  | OFF |  |  |  |  |  |
| 230 |  |  | OFF |  |  |  |  |
| 260 |  |  |  | OFF |  |  |  |
| 270 |  |  | ON |  |  |  |  |
| 280 |  | ON |  |  |  |  |  |
| 330 |  | OFF |  |  |  |  |  |
| 337 |  |  |  | ON |  |  |  |
| 340 |  |  | OFF |  |  |  |  |
| 380 |  |  | ON |  |  |  |  |
| 390 |  | ON |  |  |  |  |  |
| 392 |  |  |  | OFF |  |  |  |
| 440 |  | OFF |  |  |  |  |  |
| 447 |  |  |  | ON |  |  |  |
| 450 |  |  | OFF |  |  |  |  |
| 490 |  |  | ON |  |  |  |  |
| 500 |  | ON |  |  |  |  |  |
| 502 |  |  |  | OFF |  |  |  |
| 550 |  | OFF |  |  |  |  |  |
| 557 |  |  |  | ON |  |  |  |
| 560 |  |  | OFF |  |  |  |  |
| 600 |  |  | ON |  |  |  |  |
| 610 |  | ON |  |  |  |  |  |
| 612 |  |  |  | OFF |  |  |  |
| 660 |  | OFF |  |  |  |  |  |
| 667 |  |  |  | ON |  |  |  |
| 670 |  |  | OFF |  |  |  |  |
| 710 |  |  | ON |  |  |  |  |
| 720 |  | ON |  |  |  |  |  |
| 722 |  |  |  | OFF |  |  |  |
| 770 |  | OFF |  |  |  |  |  |
| 777 |  |  |  | ON |  |  |  |
| 780 |  |  | OFF |  |  |  |  |
| 820 |  |  | ON |  |  |  |  |
| 830 |  | ON |  |  |  |  |  |
| 832 |  |  |  | OFF |  |  |  |
| 880 |  | OFF |  |  |  |  |  |
| 887 |  |  |  | ON |  |  |  |
| 890 |  |  | OFF |  |  |  |  |
| 930 |  |  | ON |  |  |  |  |
| 940 |  | ON |  |  |  |  |  |
| 942 |  |  |  | OFF |  |  |  |
| 990 |  | OFF |  |  |  |  |  |
| 997 |  |  |  | ON |  |  |  |
| 1000 |  |  | OFF |  |  |  |  |
| 1040 |  |  | ON |  |  |  |  |
| 1050 |  | ON |  |  |  |  |  |
| 1052 |  |  |  | OFF |  |  |  |
| 1100 |  | OFF |  |  |  |  |  |
| 1107 |  |  |  | ON |  |  |  |
| 1110 |  |  | OFF |  |  |  |  |
| 1162 |  |  |  | OFF |  |  |  |
| 1217 |  |  |  | ON |  |  |  |
| 1272 |  |  |  | OFF |  |  |  |
| 1327 |  |  |  | ON |  |  |  |
| 1382 |  |  |  | OFF |  |  |  |
| 1417 |  |  |  | ON |  |  |  |
| 1430 | ON |  |  |  |  |  |  |
| 1440 | OFF |  |  |  |  |  |  |
| 1462 |  |  |  | OFF | ON | ON | ON |
| 1470 | ON |  |  |  |  |  |  |
| 1480 | OFF |  |  |  |  |  |  |
| 1530 | ON |  |  |  |  |  |  |
| 1550 | OFF |  |  |  |  |  |  |
| 1570 | ON |  |  |  |  |  |  |
| 1590 | OFF |  |  |  |  |  |  |

## Peak detection parameters and isotope ratio calculation

The parameters below can be entered in the used IRMS method of the Isodat software similar to the Time Events presented in Table S1.

Peak Detection tab:

Start/End Slope: 1.2/2.4 mV s^-1^

Background Type: “individual BGD”

History: 2 s

Evaluation tab:

Evaluation Type: CO2_SSH

Ref. Time: 105; Ref. Name “co2 working gas”; d13C/12C: -4.29; vs.: VPDB; d 18O/16O: -12.27; vs.: VPDB

The latter show the working gas values as presented in the main document and have to be set individually for each lab.

## Non-linearity of the signal intensity and δ-value of the IRMS detector

Table S2. Regression parameters for the δ-value versus mass 44 amplitude IRMS detector non-linearity.

| Regression type | Intercept (‰) | Slope (‰ mV^-1^, ‰ mV^-2^) | R² |
| --- | --- | --- | --- |
| linear | 0.374 | -0.0000599 | 0.913 |
| quadratic | 0.665 | -0.000171, 9.18e-09 | 0.994 |


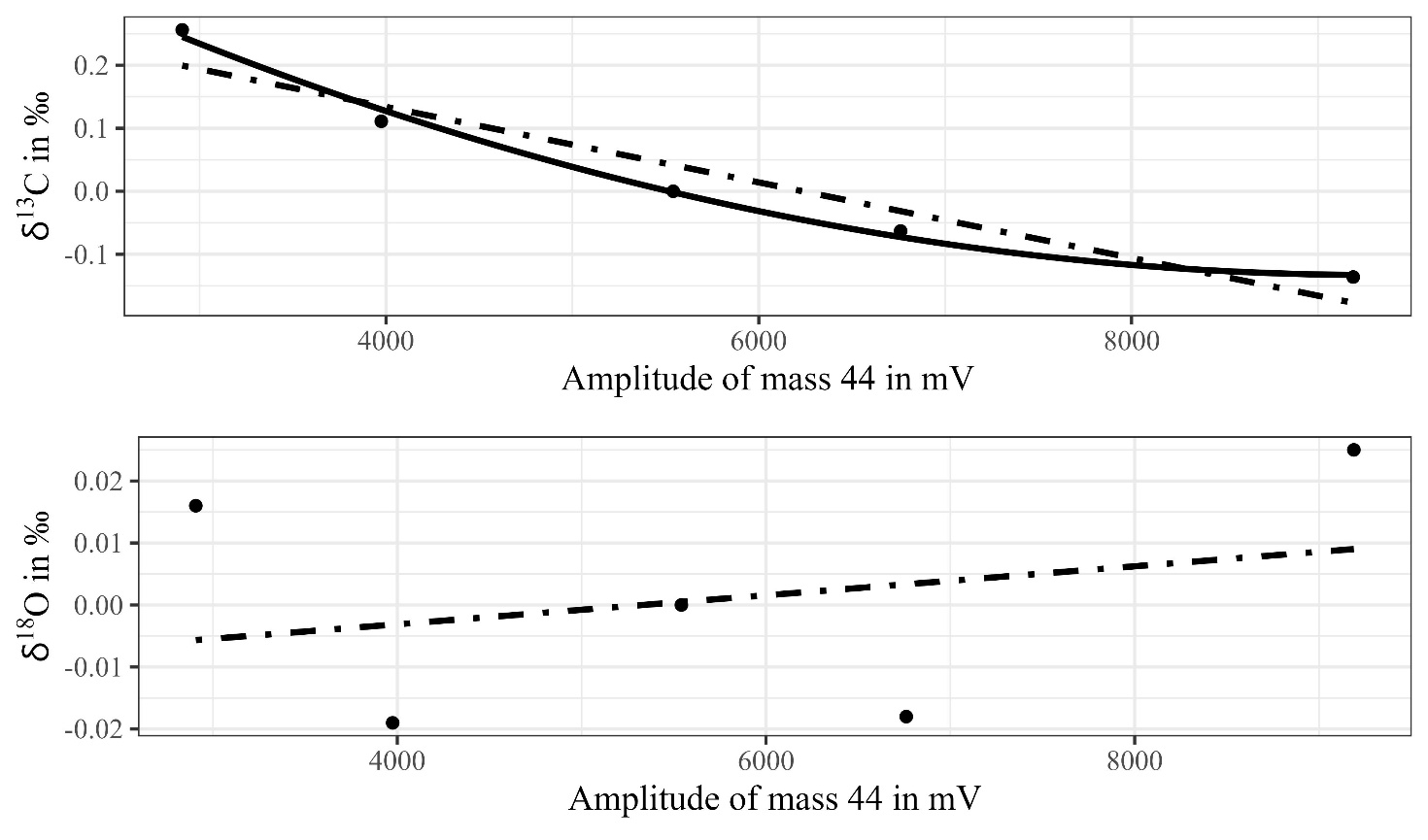


Figure S1: Plots of the IRMS-detector non-linearity of the δ-value and mass 44 amplitudes for carbon (top) and oxygen (bottom).


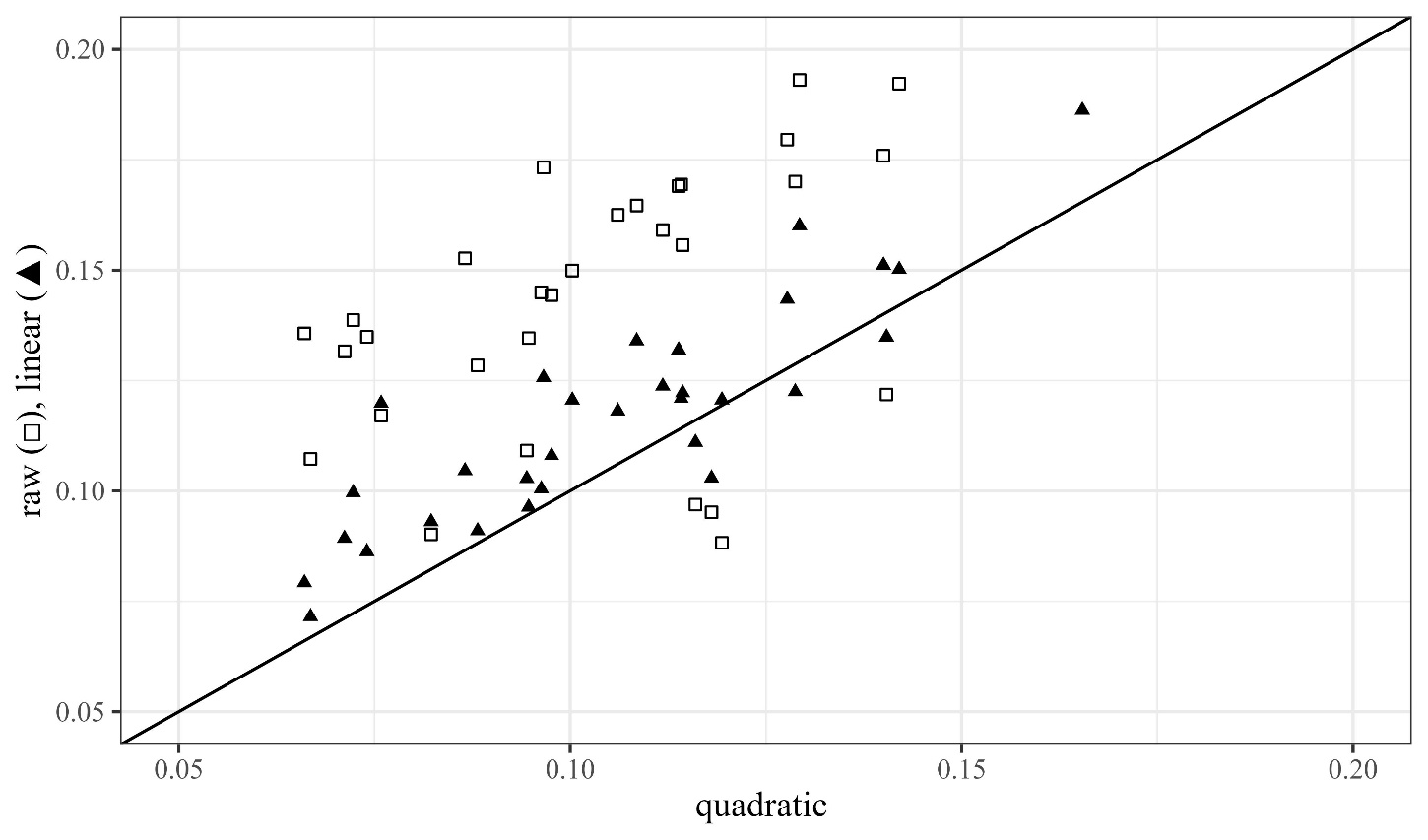


Figure S2: Comparison of the single standard deviation (1σ) of the δ^13^C values obtained from ten CO_2_ peaks per sample dependent on the type of linearity correction applied to raw δ^13^C values. Points shaped as triangles show the difference between linearity corrected values using quadratic (x axis) and linear regression (y axis). Points shaped as rectangles show the comparison of 1σ calculated from raw δ^13^C values (y-axis) vs quadratic regression corrected δ^13^C values (x-axis).


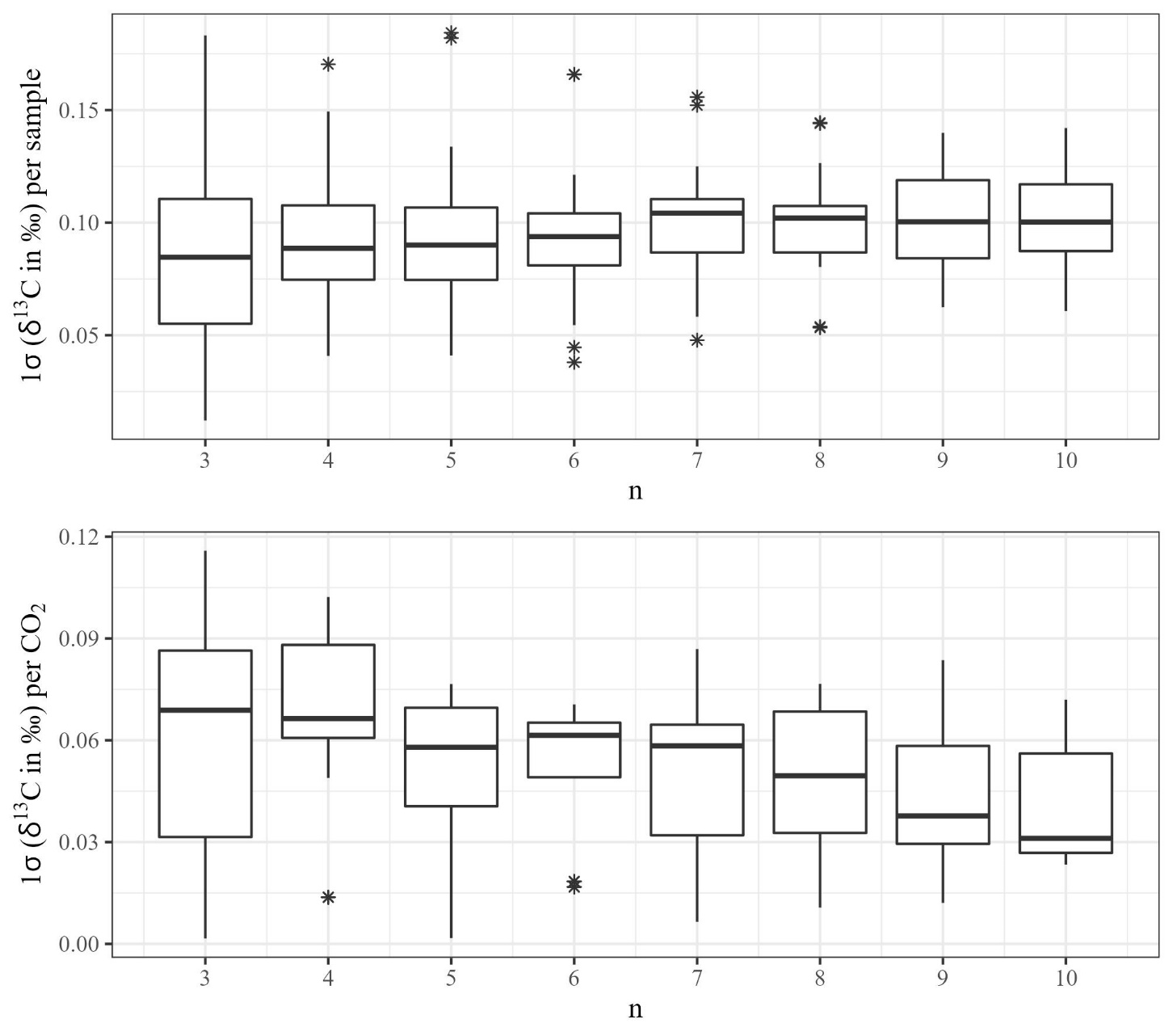


Figure S 3: Single standard deviation (1σ) of individual sample vials (top) and samples grouped by CO_2_-source (bottom) dependent on the number of CO_2_ measurement peaks used in the calculation of 1σ of carbon isotope ratios (δ^13^C) of CO_2_ in air.


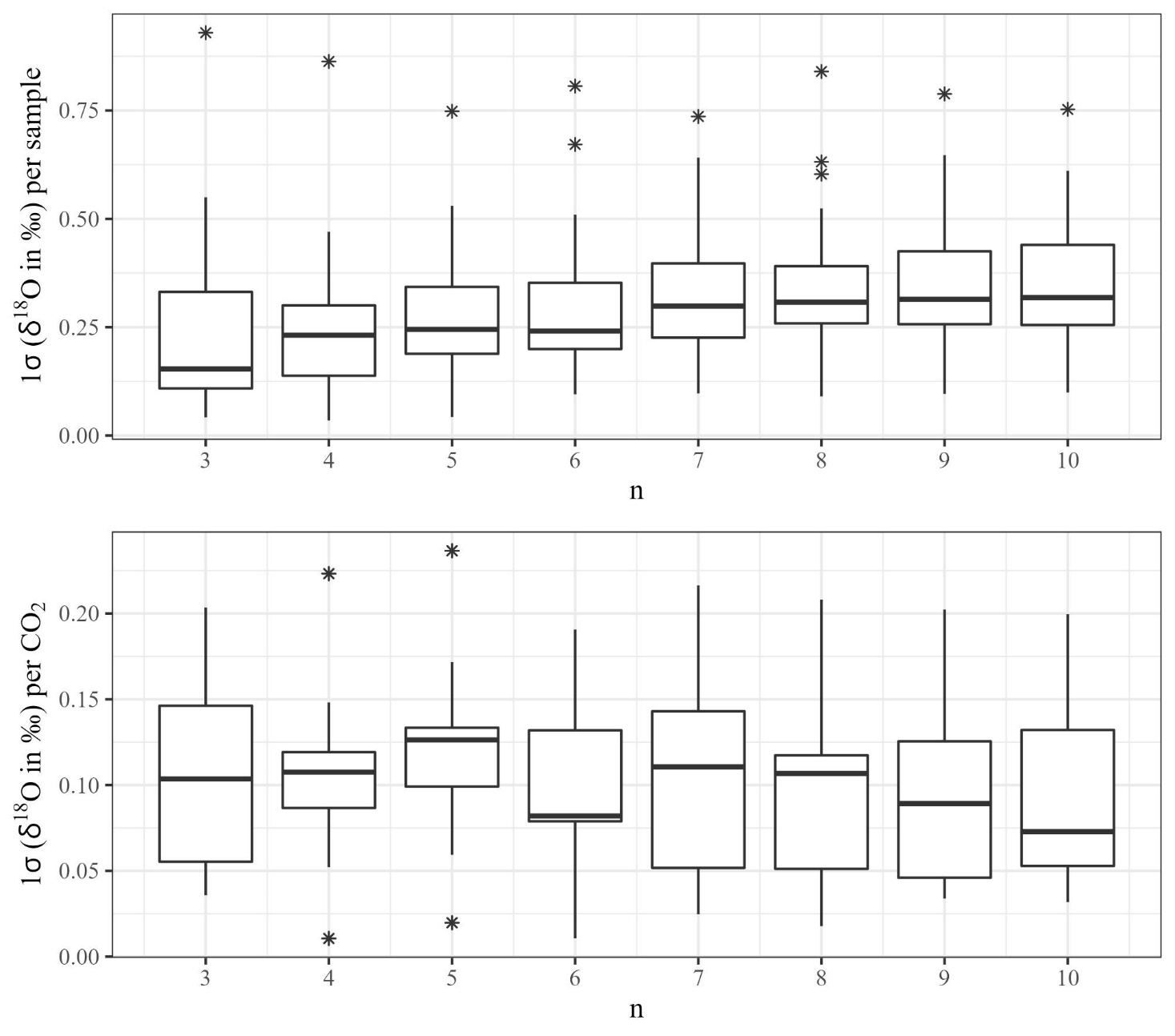


Figure S 4: Single standard deviation (1σ) of individual sample vials (top) and samples grouped by CO_2_-source (bottom) dependent on the number of CO_2_ measurement peaks used in the calculation of 1σ of the mean oxygen isotope ratio (δ^18^O) of CO_2_ in air.
